# Ramadan Fasting and Changes in Thyroid Function in Hypothyroidism: Identifying Patients at Risk

**DOI:** 10.1089/thy.2021.0512

**Published:** 2022-04-11

**Authors:** Budour Alkaf, Mohsin Siddiqui, Tomader Ali, Ali Bakir, Kevin Murphy, Karim Meeran, Nader Lessan

**Affiliations:** ^1^ Research Department, Imperial College London Diabetes Centre, Abu Dhabi, United Arab Emirates.; ^2^ Section of Endocrinology and Investigative Medicine, Imperial College London, London, United Kingdom.

**Keywords:** hypothyroidism, levothyroxine, Ramadan

## Abstract

**Background:**

Ramadan fasting (RF) is associated with major changes in meal times. This can affect thyroxine absorption and thyroid function (TF) in patients with hypothyroidism. We aimed to examine the short- and long-term impact of RF on TF in patients with primary hypothyroidism on levothyroxine.

**Methods:**

TF tests in patients with primary hypothyroidism attending an endocrine center in the United Arab Emirates were retrospectively analyzed. The impact of RF on TF, namely serum thyrotropin (TSH) TSH, free thyroxine (fT4) and free triiodothyronine (fT3), was investigated in 481 patients within 3 months before Ramadan (BR), 1–2 weeks (PR1), and 3–6 months (PR2) post-Ramadan. Controlled TF was defined as TSH between 0.45 and 4.5 μIU/mL. Inadequate control was defined as TSH >4.5 μIU/mL. Loss of control was defined as having controlled TF at BR and inadequate control at PR1. Multivariable regression analyses were used to assess the association of baseline TSH, baseline levothyroxine dose, and medication use with loss of thyroid control in Ramadan.

**Results:**

TSH increased significantly from a median of 2.0 (0.8–3.7) μIU/mL at BR to 2.9 (1.4–5.6) μIU/mL at PR1 (*p* < 0.001). This was accompanied by a fall in fT4 and fT3 at PR1 (*p* < 0.001). 25.5% of patients with previously controlled TF at BR had deterioration in TF at PR1. Sixty-one percent of patients with previously uncontrolled TF at BR remained uncontrolled at PR1. Baseline TSH was significantly associated with loss of thyroid control in Ramadan with an odds ratio (95% confidence interval) of 1.5 (1.17–1.92) (*p* < 0.001), whereas other variables, including medications known to affect levothyroxine absorption were not associated with loss of control. TSH, fT4, and fT3 levels returned to normal at PR2.

**Conclusions:**

RF can negatively affect TF of patients on levothyroxine replacement. Although this effect is modest and transitory in most patients, a significant minority exhibit more pronounced, and clinically relevant changes. The latter includes those with higher TSH BR, and a smaller group whose thyroid disease appears to be particularly affected by the mealtime and lifestyle changes of Ramadan.

## Introduction

Ramadan Fasting (RF) entails refraining from eating and drinking between dawn and sunset. It is a religious obligation widely practiced by Muslims for one lunar month a year. Although the sick are religiously exempt from the practice of RF, many choose to fast for cultural, social, personal as well as religious reasons. With the changes in meal patterns and sleeping times, physiological changes occur with RF (1–3). The long gap between main meals also dictates changes in medication timing (4). This has implications in many chronic conditions, including hypothyroidism. Muslims with hypothyroidism usually opt to fast during Ramadan (5).

Levothyroxine is the standard treatment for hypothyroidism and is primarily absorbed in the small intestine, particularly through the ileum (6). Timing of levothyroxine ingestion is also known to affect its absorption (7). Based on its pharmacokinetics, the standard recommendation is that levothyroxine should be taken on an empty stomach at least 30–60 minutes before breakfast or 3 or more hours after the evening meal (usually at bedtime) (8,9). This can be a challenge during RF. There is no breakfast as such during Ramadan; the equivalent is the predawn meal (*suhoor*).

Dinner is replaced by the sunset meal (*iftar*). It is an important social occasion and a time of intense hunger. Waking up half an hour before the planned predawn *suhoor* meal for levothyroxine administration is undesirable to most patients. Similarly, at sunset, waiting 30 minutes to an hour after levothyroxine administration to eat the sunset time (*iftar*) meal would be even less practical. Therefore, ensuring good control of hypothyroidism during the month of Ramadan is both pharmacologically and practically challenging (10–12).

**FIG. 1.**
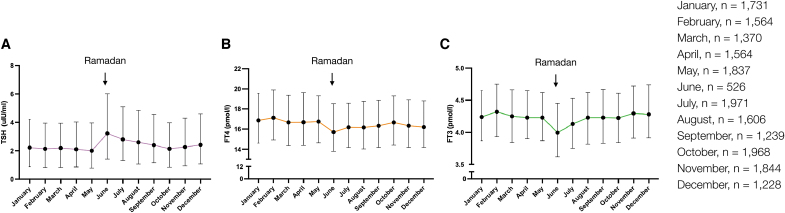
Monthly changes in TF in patients with treated primary hypothyroidism throughout a full year. Line graphs demonstrating monthly changes in median (**A**) TSH, (**B**) fT4, and (**C**) fT3 levels of 18,448 TF tests performed in patients with treated primary hypothyroidism throughout 2017. The 12 months (January–December) are labeled in the *x*-axis. The error bars represent the IQRs. In 2017, the month of Ramadan started on the 26th of May and ended on the 24th of June. For each month (January–December), the number of TF tests (*n*) were indicated. BR, before Ramadan; fT3, free triiodothyronine; fT4, free thyroxine; IQRs, interquartile range; TF, thyroid function; TSH, thyrotropin.

Although RF is widely practiced by the global Muslim population of 1.9 billion, data on the effect of RF on thyroid function (TF) are sparse, with studies investigating varied patient populations for different time periods. A few small studies have reported worsening of TF tests in patients with hypothyroidism after Ramadan (13–16). Our own data of all TF tests (*n* = 18448) performed on patients with primary hypothyroidism attending our center during 2017 showed a modest rise in thyrotropin (TSH) and a reduction in free thyroxine (fT4), and free triiodothyronine (fT3) corresponding to Ramadan period (Fig. 1). The primary objective of this study was to examine the impact of RF on variations in TF in a large cohort of patients with primary hypothyroidism on levothyroxine.

## Methods

Serum TSH, fT4, and fT3 were measured using electrochemiluminescence immunoassay (Cobas e601 platform; Roche Diagnostics). Normal reference ranges were 0.45–4.5 μIU/mL for TSH, 10.6–22.8 pmol/L for fT4, and 3.1–6.8 pmol/L for fT3.

**FIG. 2.**
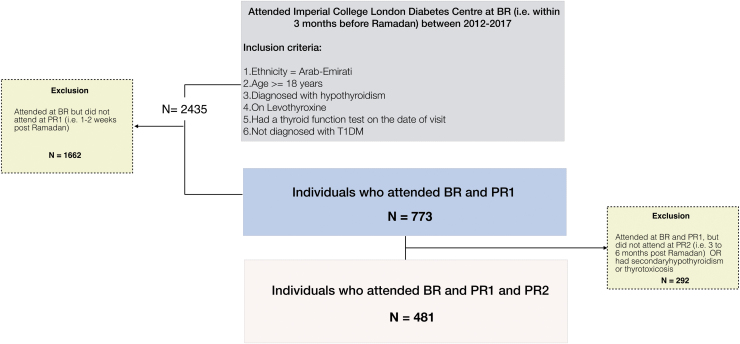
Participant flow. Flowchart demonstrating data retrieval and processing used to investigate the short-term (one to two weeks; PR1) and long-term (three to six months; PR2) impact of RF on hypothyroidism control in 481 patients with treated primary hypothyroidism. RF, Ramadan fasting.

Figure 2 summarizes the participant flow. Data on 2435 patients who attended Imperial College London Diabetes Centre (ICLDC), United Arab Emirates (UAE) between 2012 and 2017 within 3 months before Ramadan (BR) were retrieved.

### Ethics approval and consent

This study followed the ethical guidelines for retrospective studies, approved by the Medical Research Ethics Committee of ICLDC. Informed consent for use of clinical data in an anonymized form was obtained from patients at the time of enrollment.

### Study population

Patients were included based on the following criteria: (i) age ≥18 years; (ii) Arab-Emirati; (iii) diagnosed with primary hypothyroidism and on levothyroxine replacement; and (iv) had TF tests within 3 months BR, at 1–2 weeks (PR1), and 3–6 months (PR2) post-Ramadan of the same year. Where participant's TF were available for more than one study year, only the most recent data on TF and levothyroxine dose was included in the study.

Clinician's notes on fasting during Ramadan and compliance were reviewed. Pregnancy status for women at the three time points was extracted. Although trimester-specific TF ranges in UAE pregnant women on Cobas e601 platform have been previously reported in 2018 (17), these were not implemented in the center during the study period. Hence, the same assay-specific ranges have been used in pregnant women as in the nonpregnant population.

Prescribing information on levothyroxine dose and drugs that may affect levothyroxine absorption, such as proton pump inhibitors, iron and calcium supplements, was also retrieved. Mean daily dose was calculated by averaging the total dose over the week. The standard recommendation for timing of levothyroxine during Ramadan by ICLDC physicians is to take it late at night, unless half an hour before *iftar* is practical.

### Exclusion criteria

Patients with type 1 diabetes (T1DM) were excluded as they are often advised not to fast during Ramadan. Patients were also excluded if they had secondary (central) hypothyroidism or subclinical hypothyroidism.

### Statistical analysis

Statistical analyses were performed using GraphPad Prism version 8 (GraphPad Software, La Jolla, CA) and SPSS version 25 (IBM Corp., Armonk, NY). Values are presented as means with standard deviations (SD) and medians with interquartile range (IQR). The normality of parameters studied was tested using the Shapiro–Wilk test. Nonparametric paired sample tests (Wilcoxon) were used to test for differences in TSH, fT4, and fT3 values BR compared with each time point post-Ramadan (PR1 and PR2). Sensitivity analyses were performed to assess the confounding effect of pregnancy on TF. Controlled TF was defined as TSH between 0.45 and 4.5 μIU/mL and inadequate control was defined as TSH >4.5 μIU/mL. Loss of control was defined as having controlled TF at BR and inadequate control at PR1. Multivariable regression analyses were used to evaluate any potential association of age, sex, TSH and fT4 at BR, levothyroxine dose at BR, and medication use with loss of thyroid control in Ramadan.

## Results

Study population and baseline characteristics have been summarized in Table 1. Four hundred eighty-one patients with full TF tests before (BR) and after (PR1 and PR2) Ramadan were included in the study. Mean (SD) age was 44.0 (12.7) years and mean (SD) body mass index (BMI) was 30.0 (6.0) kg/m^2^. 89.9% were females, of which 14.4% (*n* = 62) were pregnant during the study. The most frequent cause of primary hypothyroidism in the study population was chronic (autoimmune) thyroiditis (88.4%). Sixteen patients (3.3%) had history of thyroid carcinoma and underwent total thyroidectomy and radioiodine ablation. Forty-seven patients (10%) had type 2 diabetes (T2DM).

**Table 1.** Basic Characteristics of Population Studied at Before Ramadan Time Point

	*N* = 481
Age (years)	
Mean (SD)	44.0 (12.7)
Median (IQR)	42.0 (35.0–52.0)
Sex, *n* (%)	
Female	432 (89.8)
Male	49 (10.2)
Indication for levothyroxine, *n* (%)	
Chronic (autoimmune) thyroiditis	422 (88.4)
Postsurgical	25 (5.2)
Total thyroidectomy and radioiodine ablation for thyroid carcinoma	16 (3.3)
Radioiodine ablation	8 (1.6)
Postpartum thyroiditis	6 (1.2)
Pregnancy, *n* (%)	
Pregnant women	62 (14.4)
Diabetes, *n* (%)	
Normal glucose tolerance	271 (56.3)
Prediabetes	149 (31.0)
T2DM	47 (9.8)
BMI (kg/m^2^)	
Mean (SD)	30.0 (6.0)
Median (IQR)	29.4 (25.7–33.3)
Lipid profile	
TC (mmol/L)	
Mean (SD)	4.7 (0.9)
Median (IQR)	4.7 (4.1–5.3)
LDL (mmol/L)	
Mean (SD)	3.1 (0.8)
Median (IQR)	3.0 (2.5–3.6)
HDL (mmol/L)	
Mean (SD)	1.4 (0.4)
Median (IQR)	1.4 (1.2–1.7)
Triglycerides (mmol/L)	
Mean (SD)	1.2 (0.6)
Median (IQR)	1.1 (0.8–1.5)
Thyroid function	
TSH (μIU/mL)	
Mean (SD)	3.7 (9.2)
Median (IQR)	2.0 (0.8–3.7)
fT4 (pmol/L)	
Mean (SD)	17.1 (4.1)
Median (IQR)	16.9 (14.6–19.3)
fT3 (pmol/L)	
Mean (SD)	4.3 (0.8)
Median (IQR)	4.2 (3.9–4.6)
Levothyroxine dose (mg)	
Mean (SD)	85.57 (42.4)
Median (IQR)	78.57 (50–100)
Interfering medications, *n* (%)	
Iron supplements	55 (11.4%)
Calcium supplements	45 (9.4%)
Proton pump inhibitors	34 (7.1%)

Data are shown as *n* (%) or mean (SD) and median (IQR).

BMI, body mass index; fT3, free triiodothyronine; fT4, free thyroxine; HDL, high-density lipoprotein; IQR, interquartile range; LDL, low-density lipoprotein; SD, standard deviations; T2DM, type 2 diabetes; TC, total cholesterol; TSH, thyrotropin.

**FIG. 3.**
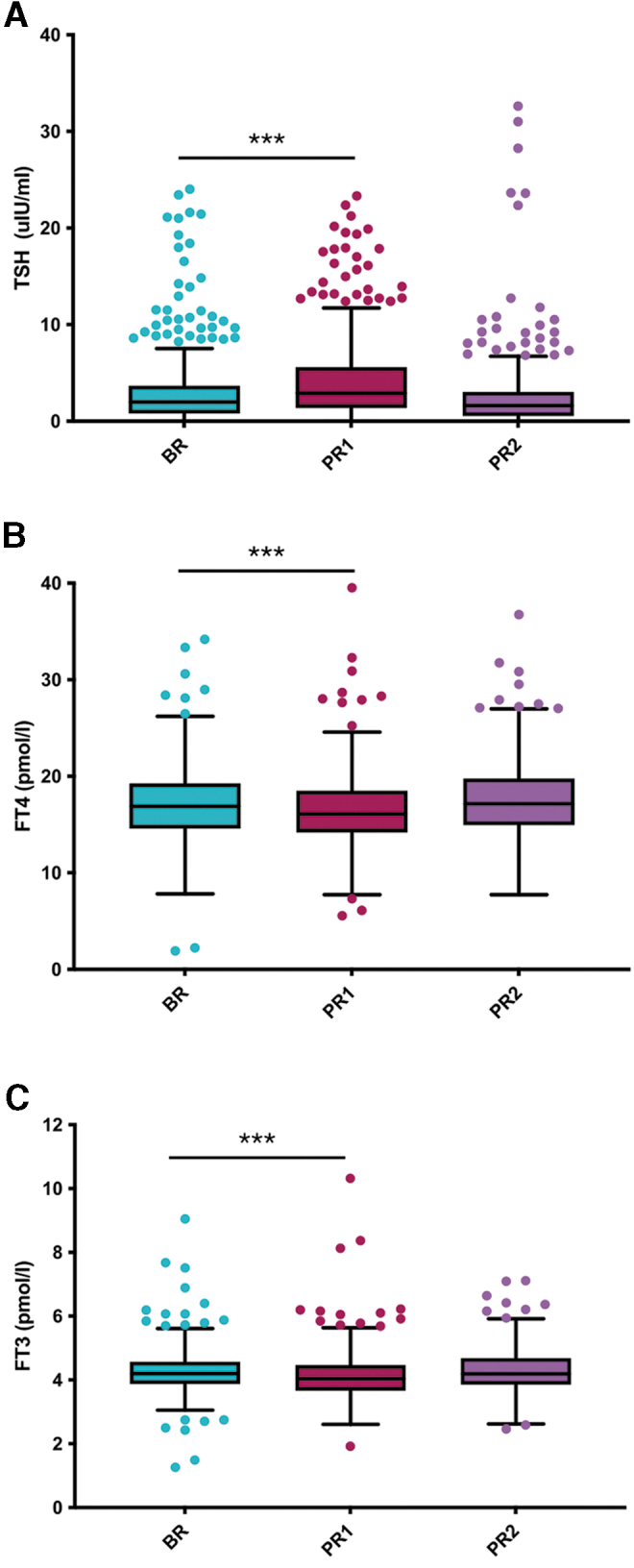
Effect of RF on TSH, fT3, and fT4 levels in patients with primary hypothyroidism. Box plots demonstrating levels **(A)** TSH, **(B)** fT4, and **(C)** fT3, at two to three months BR, one to two weeks (PR1), and three to six months post-Ramadan (PR2). Four hundred eighty-one patients with primary hypothyroidism and on levothyroxine were selected and followed up at PR1 and PR2. ***Indicates statistical significance at *p* < 0.001.

**FIG. 4.**
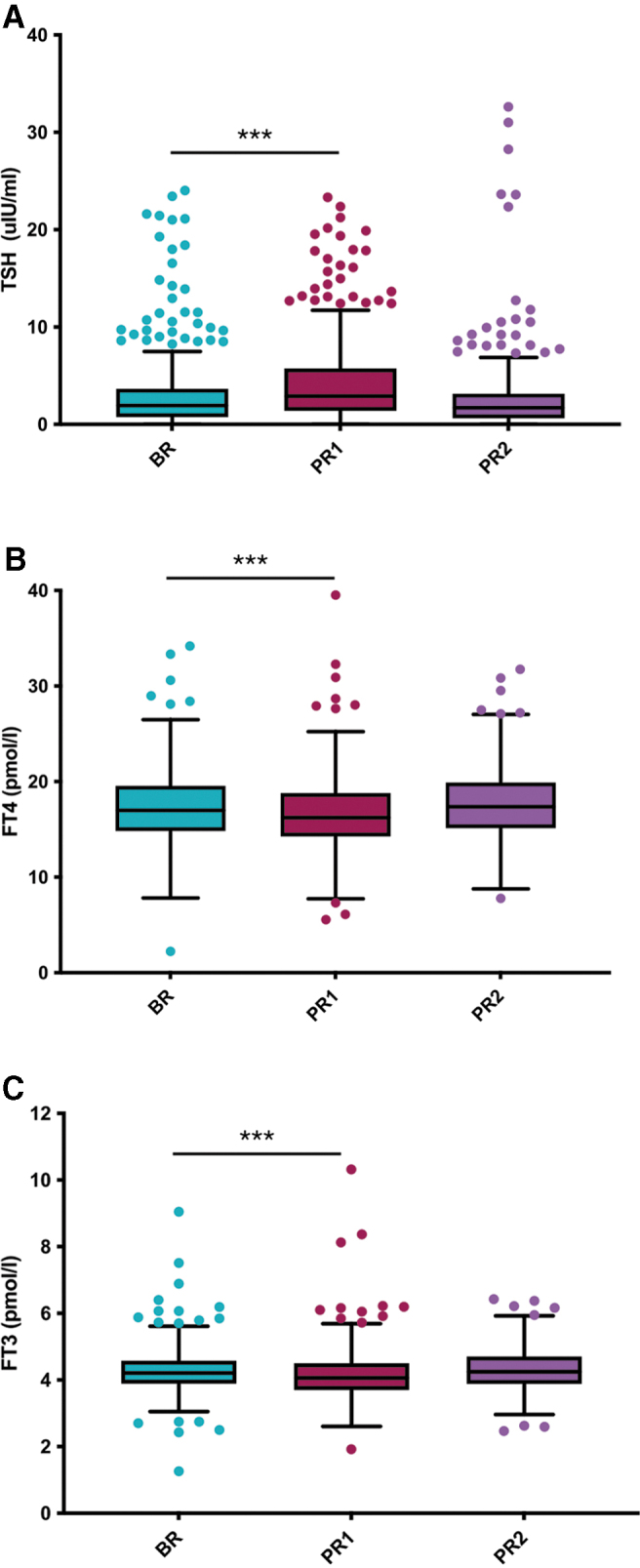
Sensitivity analysis—effect of RF on TSH, fT3, and fT4 levels in patients with primary hypothyroidism. Box plots demonstrating levels **(A)** TSH, **(B)** fT4, and **(C)** fT3, at two to three months BR, one to two weeks (PR1), and three to six months post-Ramadan (PR2). Four hundred nineteen patients with primary hypothyroidism and on levothyroxine were selected and followed up at PR1 and PR2. Data presented exclude 62 women who were pregnant at BR and/or PR1. ***Indicates statistical significance at *p* < 0.001.

TSH increased within 2 weeks post-Ramadan (PR1), from a median (IQR) of 2.0 (0.8–3.7) μIU/mL at BR to 2.9 (1.4–5.6) μIU/mL at PR1 (*p* < 0.001) (Fig. 3A). This was accompanied by a drop in fT4 and fT3, from 16.9 (14.6–19.3) and 4.2 (3.9–4.6) pmol/L at BR, to 16.1 (14.2–18.5) and 4.0 (3.7–4.5) pmol/L at PR1, respectively (*p* < 0.001) (Fig. 3B, C). At 3–6 months post-Ramadan (PR2), TSH levels dropped to a median (IQR) of 1.6 (0.5–3.0) μIU/mL. This was accompanied by a rise in fT4 and fT3 levels at PR2 to a median (IQR) of 17.1 (15.0–19.8) and 4.2 (3.9–4.7) pmol/L (Fig. 3). Sensitivity analyses did not reveal an effect of pregnancy on these observations (Fig. 4). In addition, similar results were also observed in a subgroup analysis of nonpregnant premenopausal and menopausal women, with significantly higher TSH at PR1 compared with BR in both groups (Supplementary Table S1). In men, however, no significant changes were observed (Supplementary Table S1).

In 80 patients with previously normal TF, there was a rise in TSH to above normal range within 2 weeks after Ramadan (PR1). This constituted 16.6% of the total cohort and 5.4% of patients with previously good control at BR (*n* = 314). Out of these, 52 participants had a rise in TSH above 10.0 μIU/mL. Total number of patients with TSH above normal range at BR was 93 (19.3%); 61% of this group (*n* = 57) remained inadequately controlled with TSH remaining above 4.5 mIU/L at PR1 (Supplementary Fig. S1). Out of the 481 patients, 306 (63.6%) did not have any changes in their levothyroxine dose throughout the study period.

TSH levels in this subgroup (*n* = 306) increased significantly from a median (IQR) of 1.8 (0.8–2.8) to 2.6 (1.3–4.5) (*p* < 0.001). One hundred nine patients had a change in levothyroxine dose on review at BR; 83 patients of these had dose up-titration (Supplementary Fig. S1). In this subgroup (*n* = 109), increase in TSH levels post-Ramadan was not significant [median (IQR) at BR 3.6 (0.8–7.13) vs. 3.8 (1.7–6.9) at PR1, *p* = 0.371]. There were no significant changes in weight, BMI, or lipid profile between BR and PR1 (Supplementary Table S2). The changes remained insignificant in the subgroup who were euthyroid at BR and hypothyroid at PR1 (EH).

Baseline TSH was significantly associated with loss of thyroid control in Ramadan with an odds ratio (95% confidence interval) of 1.5 (1.17–1.92) (*p* < 0.001) (Table 2). In a subset of patients who were euthyroid at BR (*n* = 314), this association remained significant (odds ratio 1.5, *p* = 0.001). However, age, sex, fT4 at BR, and medications known to affect levothyroxine absorption were not associated with loss of control.

**Table 2.** Multivariable Logistic Regression Analysis to Assess the Association of Baseline Thyrotropin, Levothyroxine Dose, and Free Thyroxine with Risk of Loss of Thyroid Control in Ramadan

Covariates	OR (CI)	*p*
BR TSH		
Unadjusted	1.5 (1.17–1.91)	0.001
Adjusted^a^	1.5 (1.17–1.92)	0.001
BR fT4		
Unadjusted	1.06 (0.97–1.15)	0.192
Adjusted^a^	1.06 (0.97–1.15)	0.207
BR levothyroxine dose		
Unadjusted	1.0051 (0.9971–1.0131)	0.209
Adjusted^a^	1.006 (0.9979–1.0142)	0.147
Interfering medications		
Unadjusted	1.13 (0.61–2.07)	0.701
Adjusted^a^	1.13 (0.60–2.13)	0.710

Four hundred eighty-one patients with primary hypothyroidism and on levothyroxine who attended at BR (two to three months BR), PR1 (one to two weeks post-Ramadan), and PR2 (three to six months post-Ramadan) were selected.

^a^ Adjusted for age and BMI at baseline (two to three months BR).

BR, before Ramadan; CI, 95% confidence interval.

## Discussion

This study demonstrates important alterations in TF in RF adults with primary hypothyroidism on levothyroxine replacement and explores factors that could explain these observations.

Our findings are consistent with other studies that have reported worsening of TF in hypothyroid patients after Ramadan (10–15). There is only one published study on the effect of RF on TF in healthy adults (*n* = 41) (13), which reported a significant gradual rise in TSH throughout the fasting month, but no significant change in fT4 and fT3 levels (13). Karoli *et al.* observed a mean TSH rise >2 mIU/L in 60% of euthyroid patients (*n* = 47), who were instructed to take levothyroxine at bedtime, at least 2 hours after the last meal (14). A prospective cohort study in 64 individuals with primary hypothyroidism showed similar results with the mean rise in TSH of 2.32 ± 3.80 mIU/L after Ramadan. The change in TSH was not affected by the timing of levothyroxine administration and time interval from the meal (10). Another prospective study on 36 Iranian hypothyroid women reported a significant drop in fT4 and a nonsignificant rise in TSH during Ramadan (15).

Interaction with interfering drugs and food, decreased compliance or physiological effects of RF on thyroid hormone secretion were considered. In our cohort, potentially interfering drugs do not appear to be a significant factor in affecting TFs during Ramadan.

Drug compliance during Ramadan takes a different form from nonfasting days as concordance with medications is frequently compromised during Ramadan. In a study comparing levothyroxine intake in two groups, 30 minutes before *iftar* and 30 minutes before *suhoor*, patients in both groups found both regimens challenging and compliance to administration timing was only 30–35% (16). In a recent randomized controlled trial by El-Kaissi *et al.* comparing three different groups who were instructed to take levothyroxine at a designated time; 30 minutes before *iftar*, ≥3 hours after *iftar*, and 30 minutes before *suhoor*, participant compliance was only between 59.3% and 77.1% (18).

Although the authors reported that the levothyroxine intake 30 minutes before *iftar* meal minimizes the TSH excursions post-Ramadan, the patient satisfaction of this intake time was moderate and the likelihood of patient concordance in routine practice is expected to be even lower. However, it is noteworthy that there was an unexplained discrepancy in the compliance and satisfaction rates among their patients; although the group taking levothyroxine in *suhoor* was most dissatisfied with that time, they remained the most compliant of the three groups. The standard recommendation by ICLDC physicians to patients for the timing of levothyroxine intake during Ramadan has been at bedtime a few hours after the *iftar* meal as it appears to be more practical and preferable for most patients.

Our data demonstrate that 60% of patients with previously inadequate control BR remain inadequately controlled with TSH above 4.5 mIU/L post-Ramadan (Supplementary Fig. S1). However, the effect of RF on changes in TSH in this subgroup in the absence of nonfasting controls remains unclear and requires further exploration in a prospective design with a comparison group. Interestingly, ∼25% of patients who were previously euthyroid lost control during Ramadan (Supplementary Fig. S1).

As such, this study identifies the less well-controlled group BR as a target group for a focused pre-Ramadan education. It is likely that these represent people with inadequate drug compliance and those with more recently diagnosed hypothyroidism. Compliance issues can also be exacerbated by the time constraints of Ramadan. In the UAE for instance, as Ramadan was in the summer months between 2012 and 2017, a typical fasting day would last for ∼14 hours (17,19). Hence, Muslims were only able to eat or drink for a few hours after sunset. The interaction of food with levothyroxine could have reduced its absorption.

In a secondary multivariable regression analysis, only baseline TSH was a predictor for loss of control during Ramadan, whereas age, sex, baseline fT4, levothyroxine dose, and interfering medications were not found to affect thyroid control in our cohort. This is in contrast to recent study from El-Kaissi *et al.* who suggested that males and older adults are more prone to loss of control post-Ramadan (20). However, as also acknowledged by the authors, this retrospective study included only 10 male participants and yielded statistically insignificant results.

The more recent prospective study from the same investigators did not demonstrate the effect of age or sex on Ramadan-related TSH elevation (18). Our study benefits from a higher number of male participants (*n* = 49) and did not find that they are at higher risk of loss of control during Ramadan. Premenopausal women are exempted from fasting during their menstrual periods; our analysis showed a more notable and significant rise in TSH in this subgroup. Hence, the abstinence from fasting for a few days during menstruation does not appear to diminish the changes in TSH observed.

Therapeutically, small increases in levothyroxine dose by 25–50 μg/day from the beginning of Ramadan till 2–3 weeks post-Ramadan have been previously suggested to improve the control of hypothyroidism (11–16). However, the effect of this small increase in levothyroxine dose or the timing of its administration during Ramadan has not been evaluated in a prospective trial. Our data suggest that this postulated dose increase can be considered for individuals with TSH at the upper end of the normal range BR and not necessarily in all patients.

There did not appear to be any significant changes in weight, BMI, or lipid profile in patients during Ramadan. Since the observed TSH changes are relatively minor in most patients, the magnitude of their effect on lipids is unlikely to be significant. In addition, concomitant lipid lowering therapy could be an important confounding factor in these patients.

Based on our study findings, we suggest clinicians take into consideration the possibility that a patient may have just completed a period of fasting, and that physicians may need to be cautious in making any dose adjustments in the early period after Ramadan. However, this should not discourage physicians from assessing TF after Ramadan if clinically warranted.

Potential limitations of our study include selection bias and its retrospective design. The latter has allowed us to look at changes in a relatively large number of patients with hypothyroidism in the context of RF and in a “real world” setting. In this study, we deliberately chose patients who had TF data at all three time points of interest, and as such, the study may have been subject to some selection bias. In addition, the retrospective design of the study meant it was not possible to confirm whether the subjects included in the study fasted during the entire month of Ramadan in the study year.

However, studies during Ramadan in the Middle East, including those on patients with diabetes for instance (21,22) and our own experience in Emirati individuals with T1DM and T2DM in a prospective study has shown that at least 80% of these choose to fast most of Ramadan (unpublished data). Hence, extrapolating from Diabetes and Research alliance (16) surveys conducted in high-risk patients, it will be safe to assume that patients with thyroid disorders are even more likely to fast during Ramadan. Since RF is so commonly practiced among the Emirati population even with multiple comorbidities, whether a patient practiced RF was not specifically documented in clinic consultations post-Ramadan (PR1).

Another limitation is that the levothyroxine administration timing during Ramadan and its relation to meals was not available in the patient records. Changes in levothyroxine dose may warrant more frequent visits. However, only a quarter of the total cohort in our study had a dose change at BR, and thus it is unlikely that this was a major contributing factor for repeat TFs post-Ramadan. Moreover, coexisting conditions such as malabsorption syndromes, autonomic neuropathy from diabetes, or previous bariatric surgery may influence the levothyroxine absorption, but the number of people with these comorbidities was small, and as such the overall effect on TF is likely to be minor.

## Conclusions

In conclusion, we have demonstrated that RF is associated with a statistically significant rise in TSH in patients on levothyroxine treatment. However, the magnitude of the observed change in TSH in our study is not of clinical significance at a population level. In a multivariable analysis, we observed that a small group of patients with higher TSH are potentially at an increased risk for loss of TF control during Ramadan. Health care professionals should consider discussing the potential implications of RF on TF control with levothyroxine-treated patients, with special attention to those whose TF are already inadequately controlled.

Prospective studies are needed to evaluate the clinical significance of these changes. Such studies will have the advantage of specific information on the actual timing of levothyroxine ingestion and the number of days fasted by the participants. A tailored levothyroxine dose increase may be considered in some patients. Future studies will also need to explore any associated changes in physiological processes and the hypothalamic-pituitary-thyroid axis, which may impact TF control during RF.

## Supplementary Material

Supplemental data

Supplemental data

Supplemental data
