# Supplementary material for: Ramadan Fasting and Changes in Thyroid Function in Hypothyroidism: Identifying Patients at Risk
Source: Thyroid. 2022 Apr 11;32(4):368–75. doi: 10.1089/thy.2021.0512 (PMC9048180; doi:10.1089/thy.2021.0512)
Supplement: Supplemental data [file Supp_TableS1.docx]

| **Supplementary Table 1.** Subgroup analysis on the impact of Ramadan on TSH levels men, premenopausal women, and menopausal women with primary hypothyroidism and on levothyroxine. | | | |
| --- | --- | --- | --- |
|  | Premenopausal women | | |
| N | 261 | | |
|  | BR | PR1 | *P* |
| TSH (uIU/ml) | 1.81 (0.71-3.56) | 3.01 (1.39-5.55) | <0.001 |
|  | Menopausal women | | |
|  | 109 | | |
|  | BR | PR1 | *P* |
| TSH (uIU/ml) | 1.92 (0.73-3.65) | 2.78 (1.31-5.97) | 0.005 |
|  | Men | | |
|  | 49 | | |
|  | BR | PR1 | *P* |
| TSH (uIU/ml) | 2.55 (1.21-4.24) | 2.90 (1.74-6.83) | 0.151 |
