# Supplementary material for: Ramadan Fasting and Changes in Thyroid Function in Hypothyroidism: Identifying Patients at Risk
Source: Thyroid. 2022 Apr 11;32(4):368–75. doi: 10.1089/thy.2021.0512 (PMC9048180; doi:10.1089/thy.2021.0512)

**Supplementary Figure 1. Flowchart demonstrating distribution of patients before and after Ramadan according to their TSH levels.** Flowchart is divided into three parts; green for before Ramadan (BR), red for 1-2 weeks post Ramadan (PR1), and purple for 3-6 months post Ramadan (PR2). Patients were categorized into three groups at BR: (1) hyperthyroid (TSH < 0.4 uIU/ml), (2) euthyroid (TSH = 0.4-4.5 uIU/ml), or (3) hypothyroid (TSH > 4.5 uIU/ml). Each group was then sub-grouped in PR1 and PR2 based on the changes in their TSH levels at each time point. Red and green arrows represent the number of patients who reported an increase or a decrease in their levothyroxine dose during Ramadan, respectively.


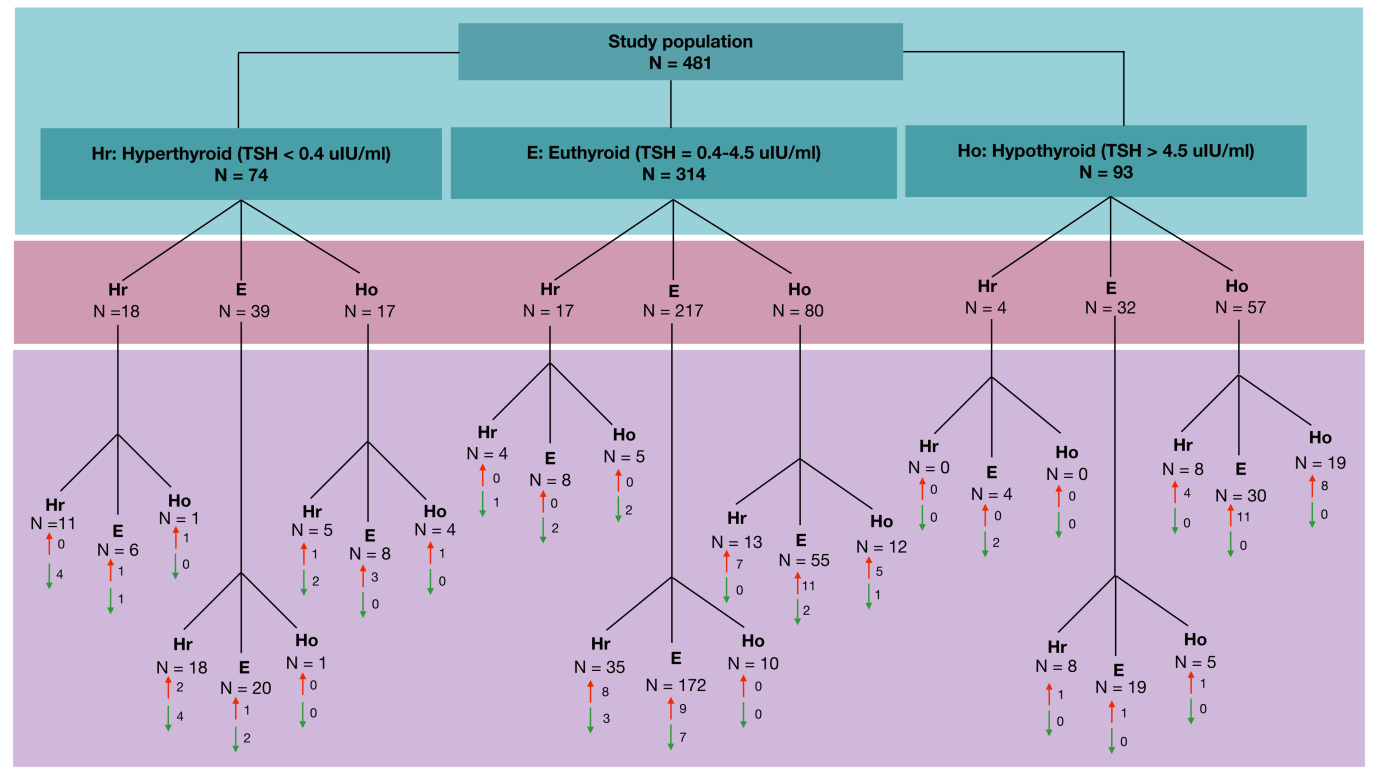

Supplement: Supplemental data [file Supp_FigS1.docx]
