# Supplementary material for: Ramadan Fasting and Changes in Thyroid Function in Hypothyroidism: Identifying Patients at Risk
Source: Thyroid. 2022 Apr 11;32(4):368–75. doi: 10.1089/thy.2021.0512 (PMC9048180; doi:10.1089/thy.2021.0512)
Supplement: Supplemental data [file Supp_TableS2.docx]

| Supplementary Table 2. Comparing lipids and weight between BR and PR1 in 481 patients categorised according to their thyroid control at BR and PR1. | | | |
| --- | --- | --- | --- |
| **All** | | | |
| **N** | **481** | | |
|  | **BR** | **PR1** | |
|  | Median (IQR) | Median (IQR) | *P* |
| Weight (kg) | 74.5 (65.0-85.5) | 73.3 (63.3-83.9) | 0.197 |
| BMI (kg/m2) | 29.4 (25.7-33.3) | 29.1 (25.5-32.9) | 0.418 |
| LDL (mmol/l) | 3.0 (2.5-3.6) | 3.1 (2.5-3.6) | 0.441 |
| HDL (mmol/l) | 1.4 (1.2-1.7) | 1.4 (1.2-1.6) | 0.315 |
| Triglycerides (mmol/l) | 1.1 (0.8-1.5) | 1.1 (0.8-1.6) | 0.575 |
| Total cholesterol (mmol/l) | 4.7 (4.1-5.3) | 4.7 (4.1-5.3) | 0.517 |
| **EE** | | | |
| **N** | **217** | | |
|  | **BR** | **PR1** | |
|  | Median (IQR) | Median (IQR) | *P* |
| Weight (kg) | 71.2 (63.2-79.1) | 73.2 (64.3-83.8) | 0.573 |
| BMI (kg/m2) | 27.6 (24.9-31.4) | 29.0 (25.7-32.7) | 0.633 |
| LDL (mmol/l) | 3.1 (2.6-3.5) | 3.1 (2.7-3.7) | 0.568 |
| HDL (mmol/l) | 1.4 (1.2-1.7) | 1.4 (1.2-1.7) | 0.767 |
| Triglycerides (mmol/l) | 1.1 (0.7-1.5) | 1.1 (0.9-1.5) | 0.500 |
| Total cholesterol (mmol/l) | 4.8 (4.3-5.3) | 4.7 (4.0-5.4) | 0.615 |
| **EH** | | | |
| **N** | **80** | |  |
|  | **BR** | **PR1** | |
|  | Median (IQR) | Median (IQR) | *P* |
| Weight (kg) | 71.2 (63.2-79.1) | 71.0 (60.8-77.2) | 0.531 |
| BMI (kg/m2) | 27.6 (24.9-31.4) | 26.8 (25.3-31.6) | 0.692 |
| LDL (mmol/l) | 3.0 (2.4-3.6) | 3.2 (2.5 - 3.6) | 0.368 |
| HDL (mmol/l) | 1.5 (1.1-1.7) | 1.3 (1.1-1.6) | 0.133 |
| Triglycerides (mmol/l) | 1.1 (0.9-1.5) | 1.3 (0.9-1.6) | 0.334 |
| Total cholesterol (mmol/l) | 4.7 (4.0-5.4) | 4.8 (4.0-5.5) | 0.476 |
| **HE** | | | |
| **N** | **32** | |  |
|  | **BR** | **PR1** | |
|  | Median (IQR) | Median (IQR) | *P* |
| Weight (kg) | 77.9 (69.1-82.9) | 73.2 (65.3-81.1) | 0.306 |
| BMI (kg/m2) | 29.8 (27.4-33.8) | 29.3 (26.6-31.6) | 0.350 |
| LDL (mmol/l) | 3.0 (2.4-3.8) | 3.1 (2.5-4.0) | 0.942 |
| HDL (mmol/l) | 1.3 (1.1-1.4) | 1.3 (1.2-1.5) | 0.895 |
| Triglycerides (mmol/l) | 1.0 (0.9-1.5) | 1.0 (0.9-1.2) | 0.971 |
| Total cholesterol (mmol/l) | 4.6 (4.1-5.5) | 4.8 (3.9-5.2) | 0.971 |
| **HH** | | | |
| **N** | **57** | |  |
|  | **BR** | **PR1** | |
|  | Median (IQR) | Median (IQR) | *P* |
| Weight (kg) | 77.7 (65.5-87.9) | 79.1 (64.9-85.5) | 0.661 |
| BMI (kg/m2) | 29.9 (26.7-34.5) | 30.9 (27.1-34.6) | 0.793 |
| LDL (mmol/l) | 2.9 (2.6-3.7) | 2.8 (2.3-3.3) | 0.340 |
| HDL (mmol/l) | 1.4 (1.1-1.7) | 1.3 (1.2-1.5) | 0.234 |
| Triglycerides (mmol/l) | 1.2 (0.9-1.8) | 1.2 (0.9-1.8) | 0.773 |
| Total cholesterol (mmol/l) | 4.6 (4.2-5.2) | 4.5 (4.0-4.9) | 0.185 |
